# NATURAL ENVIRONMENTS’ INFLUENCE ON RESPONSIVE BEHAVIOUR, WORK-RELATED STRESS, BURNOUT, AND TURNOVER INTENTION

**DOI:** 10.1093/geroni/igad104.3791

**Published:** 2023-12-21

**Authors:** Peggy Chi, Adrian Wagg, Stephen Verderber, Olesya Falenchuk, Whitney Berta

**Affiliations:** University of Toronto, Toronto, Ontario, Canada; University of Alberta, Edmonton, Alberta, Canada; University of Toronto, Toronto, Ontario, Canada; University of Toronto, Toronto, Ontario, Canada; University of Toronto, Toronto, Ontario, Canada

## Abstract

Natural environments in healthcare design are thought to promote health. However, its empirical research in long-term care (LTC) has been limited; the degree of rigor is mixed, focusing on older adults with little research on professional caregivers. This study examined the relationships between the natural environment and the mental health and well-being of older adults 65+ living with Alzheimer’s disease and related dementias (residents) and of their professional caregivers in LTC homes using a rigorously developed survey to standardize an approach to the evaluation of natural environments for these population groups. A cross-sectional, multiphase mixed method study was conducted that included a scoping review (n=137), a Delphi panel (n=24 experts), cognitive debriefing interviews (n=33), psychometric evaluations (n=83 LTC home areas), and multilevel modeling and regression analyses (n=1599 residents; n=563 professional caregivers). A conceptual framework on the natural environment in healthcare was developed, which informed the development of the Natural Environment Survey for Long-Term Care. This survey evaluates 1) the design of natural environments, 2) residents’ usage of and exposure to natural environments, and 3) professional caregivers’ usage of, exposure to, and perception of natural environments. Unlocked doors to outdoor spaces, outdoor activities, and indoor activities were significantly associated with residents’ responsive behavior in secure home areas (i.e., locked access and egress). Usage of and exposure to the natural environment during breaks and leisure were associated with their professional caregivers’ work-related stress, burnout, and turnover intention in secure and non-secure home areas. These findings have potential utility for decision-makers transforming LTC.

